# Should we offer deep brain stimulation to Parkinson’s disease patients with GBA mutations?

**DOI:** 10.3389/fneur.2023.1158977

**Published:** 2023-04-14

**Authors:** Carlo Alberto Artusi, Leonardo Lopiano

**Affiliations:** ^1^Department of Neuroscience “Rita Levi Montalcini”, University of Turin, Turin, Italy; ^2^SC Neurologia 2U, AOU Città della Salute e della Scienza, Turin, Italy

**Keywords:** Parkinson disease, GBA, genetics, deep brain stimulation, subthalamic nucleus, globus paliidus, cognition, dementia

## Abstract

Parkinson’s disease (PD) patients who are carriers of glucosylceramidase β1 (GBA1) gene mutations typically have an earlier age at onset and a more aggressive disease course, with a higher burden of neuropsychological issues. The use of deep brain stimulation (DBS) in PD patients with disabling motor fluctuations and absence of dementia is a widespread therapeutic option, often with good results in terms of improvement in activities of daily living and quality of life. Although all PD patients, when fulfilling the common selection criteria for DBS, can benefit from this intervention, some studies have raised attention toward the fact that PD patients who are carriers of GBA1 variants may have a worse DBS outcome possibly due to an accelerated progression of cognitive decline. From this viewpoint, we summarize the current literature, highlighting the knowledge gaps and proposing suggestions for further research as well as for clinical practice in this timeframe of uncertainty related to using DBS in PD patients who are carriers of GBA1 variants.

## Introduction

Parkinson’s disease (PD) has been traditionally viewed as a single idiopathic disorder. The knowledge advancement on pathophysiological mechanisms and deep phenotyping challenged this view. Specifically, advancements in genetics in the last 10 years have accelerated our PD etiopathogenic understanding and offered a biological basis to the phenotypic stratification in different PD subtypes and disease trajectories (1).

An outstanding example is the discovery that the carriers of glucosylceramidase β1 (GBA1) gene mutations typically show an earlier age at onset and a more aggressive disease course, correlated with a greater probability of developing cognitive impairment, neurobehavioral issues, REM sleep behavior disorder, and other nonmotor symptoms, as well as postural instability and falls. However, the high number of GBA1 variants and other more elusive aspects such as genetic, epigenetic, or environmental modulators may modify the penetrance, age at onset, and the course of the disease in carriers of the same mutation.

While these pieces of information can have low relevance when considering the traditional approach with dopaminergic therapies, the selection of optimal candidates for deep brain stimulation (DBS) demands a more nuanced characterization of the distinctive and heterogeneous pathogenic mechanisms involved in the different PD subtypes (2).

Specifically, some studies have raised attention to the fact that PD carriers of GBA1 variants (PD-GBA1) may have a worse DBS outcome, possibly due to an accelerated progression of cognitive decline.

## The cognitive risk of PD-GBA1 patients treated with STN-DBS

In 2012, a case series of three PD-GBA1 and six non-GBA1 carriers showed a higher prevalence of dementia in the PD-GBA1 group after 24 to 48 months of STN-DBS (3). In 2013, a 5-year prospective study found a steeper decline in Mattis Dementia Rating Scale (MDRS) scores in 13 PD-GBA1 patients compared with 67 non-GBA1 carriers (4). In that study, data from six PD-GBA1 patients were available only after 5 years; however, the mean decline in MDRS-2 scores for PD-GBA1 was 4.4 points per year compared with 0.5 points per year among non-GBA1 carriers. Interestingly, 2 of 13 PD-GBA1 underwent globus pallidus pars interna (GPi)-DBS surgery, a higher proportion than mutation-negative patients.

Successively, a 7-year follow-up study found worse performance in all of the five cognitive domains in 17 PD-GBA1 than in 17 non-GBA1 carriers matched for sex and disease duration (5).

Some reviews and a meta-analysis based on the data from the above-mentioned studies concluded that PD-GBA1 patients can achieve a significant motor benefit from DBS (although with a possibly less degree than the general PD population) but may have a fast neuropsychological decline (2, 6, 7). Whether the DBS influences this decline or is just part of the PD-GBA1 disease progression remains an open question. In 2022, a retrospective multicenter study analyzed longitudinal cognitive data of 366 PD patients with or without GBA1 mutations and treated or not with STN-DBS (8). The main finding of the study is represented by a steeper decline of the MDRS score in the group of PD-GBA1 treated with STN-DBS than in the other three groups: non-GBA1 carriers treated with DBS, non-GBA1 carriers not treated with DBS, and PD-GBA1 not treated with DBS. The study has limitations related to the retrospective study design and the absence of a control group adequately matched for the main demographic and clinical features. Moreover, the steeper decline of MDRS in PD-GBA1 treated with STN-DBS does not directly reflect the onset of mild cognitive impairment or dementia, and the clinical implication of this computational analysis remains to be clarified. Notwithstanding all these aspects, this study corroborates the warning related to the fact that the combined effects of GBA1 mutations and STN-DBS may negatively impact cognition, at least at a level group. Prospective and case–control studies with adequate follow-up and sample size are needed to confirm the negative impact of STN-DBS on the cognitive decline of PD-GBA1 patients and the possible role of this decline at a single-subject level.

## Is there a role for different GBA1 variants?

There are case reports reporting the long-lasting efficacy of STN-DBS in the absence of dementia onset in PD-GBA1 patients, even in carriers of severe variants (9, 10). This aspect is relevant considering the high number of pathogenic GBA1 variants associated with PD, accounting for a supposed differential PD phenotype according to the Gaucher disease (GD) type caused by the homozygous presence of the specific variant. In fact, GBA1 variants are currently divided into three groups: risk variant, mild, and severe. Currently, we consider mild mutations as those that cause GD type I, and severe mutations as those that cause GD types II and III (11). Severe variants like p.L444P are found to be associated with an increased PD risk, earlier age at onset, and greater cognitive dysfunction compared to less-severe mutations like p.N370S (12–14). According to a large phenotype–genotype study on PD-GBA1 published in 2016, carriers of severe mutations had a greater risk for dementia than those of mild mutations (5-fold in severe PD-GBA1 and 2-fold in mild PD-GBA1) but similar mortality risk (15). Moreover, PD-GBA1 with severe variants had a significant blood flow reduction in the bilateral parietal lobe at a perfusion single-photon emission computed tomography than PD-GBA1 with mild variants. We believe the role of variants should be taken into account when considering DBS, although the DBS effect on cognition according to the different GBA1 variants still needs to be clarified. The main clue related to the role of different variants is provided by the aforementioned retrospective and multicenter study published in 2022 (8); the study findings also suggest that there are no major differences in the cognitive decline after STN-DBS in PD-GBA1 carriers of severe or mild variants, while the carriers of risk variants could have a less degree of decline in the MDRS score than the carriers of mild or severe variants.

## The role of DBS target

Considering that the vast majority of PD-GBA1 treated with DBS, as reported in the literature, received chronic electrical stimulation of bilateral STN and given the supposed ‘cognitively safer’ action of GPi-DBS, it seems an urgent need to obtain data from PD-GBA1 treated with GPi-DBS. Indeed, the outcome comparison between GPi- and STN-DBS in the general PD population suggests that GPi-DBS has a less cognitive impact (16). The compact anatomy of STN compared with GPi renders more frequent the unintended current spread into adjacent STN subregions and nearby structures possibly interfering with cognitive functions. In particular, the unintended interference with the nonmotor medial prefrontal–striatal circuitry, activated by the stimulation of the limbic part of the small STN, can affect some cognitive functions (17). However, there is still uncertainty about how DBS can negatively impact cognition and behavior in some patients. It has been suggested that in some instances, neurosurgical implantation can be detrimental, especially in patients with presurgical cognitive impairment (18). A prospective study assessing motor and nonmotor outcomes of PD-GBA1 treated either with STN- or GPi-DBS and stratified for the different variants is warranted. As STN patients may sustain greater cognitive decline after surgery, and current clinical practice and expert consensus provide the use of GPi-DBS in PD patients with a presurgical impaired cognition, a more cautious approach to PD-GBA1 patients by targeting the GPi could be considered and discussed between the clinicians, the patient, and his/her family, awaiting further literature evidence (19).

## Discussion and future directions

Data derived from the current literature on the impact of DBS on PD-GBA1 are not conclusive, and more robust evidence is needed to achieve firm conclusions. Therefore, it is important not to deny PD-GBA1 patients the possibility to benefit from DBS only based on their genetic status but rather inform them of the potential of an accelerated cognitive decline in a comprehensive and multidimensional discussion of pros and cons of DBS, considering all personal and clinical features, the impact of motor symptoms, and the will and expectations of the patients. Particular attention should be paid to the presurgical cognitive status and also the presence of a mild vs. severe GBA1 variant, which could be a determinant for an accelerated cognitive decline (at least at a group level). This kind of informed choice would imply that all patients are genetically screened for the presence of GBA1 mutations before undergoing neurosurgery, which means an increase in costs and possible surgical delays.

Moreover, when PD-GBA1 is considered for DBS, the choice of the DBS target becomes even more relevant. In fact, considering the ‘cognitive frailty’ of these patients and waiting for further data from studies, it seems reasonable to consider the ‘cognitively safer’ GPi-DBS instead of STN-DBS, especially for patients with severe GBA1 variants. However, the lack of data on GPi-DBS in these patients makes the choice of GPi target an option to be carefully discussed in a multidisciplinary assessment including the neurologist, the neurosurgeon, and the neuropsychologist and taking into account the preference of the patient, who needs to be correctly informed about the possible risks and benefits.

Future studies could help refine these indications and hopefully reveal the pathogenic underpinnings of such cognitive issues in PD patients who are carriers of GBA1 variants. Biomarkers like the levels of glucocerebrosidase activity in the cerebrospinal fluid are promising in predicting the risk of dementia development in PD patients (with and without GBA1 variants) (20). They might also prove to be helpful in the near future in determining patients at higher risk of cognitive decline after DBS surgery.

In conclusion, the current literature does not help clarify the complex relationship between GBA1 mutations, DBS, and cognitive decline; however, many studies suggest a possible negative impact of STN-DBS on the progression of cognitive decline in PD-GBA1. Considering the available information, it seems reasonable to analyze the presence of GBA1 variants in candidates for DBS, inform carriers of the potential risks, and evaluate the possibility of targeting GPi in these patients.

## Data availability statement

The original contributions presented in the study are included in the article/Supplementary material, further inquiries can be directed to the corresponding author.

## Author contributions

CA wrote the first draft of the manuscript. CA and LL contributed to data collection. LL reviewed and critiqued the manuscript. All authors contributed to the article and approved the submitted version.

## Conflict of interest

The authors declare that the research was conducted in the absence of any commercial or financial relationships that could be construed as a potential conflict of interest.

## Publisher’s note

All claims expressed in this article are solely those of the authors and do not necessarily represent those of their affiliated organizations, or those of the publisher, the editors and the reviewers. Any product that may be evaluated in this article, or claim that may be made by its manufacturer, is not guaranteed or endorsed by the publisher.
